# Myelopathy Caused by Chronic Epidural Hematoma Associated with L1 Osteoporotic Vertebral Collapse: A Case Report and Review of the Literature

**DOI:** 10.2174/1874325000802010040

**Published:** 2008-03-26

**Authors:** Itaru Oda, Masanori Fujiya, Kyoichi Hasegawa, Satoshi Terae

**Affiliations:** 1 Hokkaido Orthopaedic Memorial Hospital, Hokkaido University Graduate School of Medicine, Sapporo, Japan.; 2 Department of Radiology, Hokkaido University Graduate School of Medicine, Sapporo, Japan.

**Keywords:** Chronic epidural hematoma, osteoporotic vertebral collapse, thoracolumbar spine.

## Abstract

Epidural hematoma associated with osteoporotic vertebral collapse has not been reported yet in the literature. We report a case of myelopathy caused by chronic epidural hematoma associated with L1 osteoporotic vertebral collapse and review the relevant literature.

## INTRODUCTION

Intracranial epidural hematoma is a common lesion, however, spinal epidural hematoma is relatively rare. Spinal epidural hematoma may result from coagulopathy, vascular lesions, iatrogenesis, spontaneous occurrence, and fresh spinal injuries [1-8]. Osteoporotic vertebral collapse is a kind of pseudarthrosis and gradually results from a vertebral fracture in the osteoporotic spine [9-11]. In the literature, to date, no case of epidural hematoma associated with osteoporotic vertebral collapse has been reported.

We present a patient with chronic epidural hematoma in the thoracic spine associated with L1 osteoporotic vertebral collapse. A literature review will follow.

## CASE REPORT

A 78-year-old female with L1 osteoporotic vertebral collapse was referred to our hospital after two months of conservative treatments by the former doctor. Her chief complaints were severe back pain, motor weakness as well as pain of bilateral lower limbs, and bladder-bowel dysfunction. She was unable to walk or keep sitting. Neurological evaluation demonstrated both myelopathy and cauda equina syndrome including sensory loss below T10 dermatome, bilateral positive Babinski reflex, hyporeflexia and motor weakness of the bilateral lower extremities, and neurogenic bladder.

**Fig. (1):**
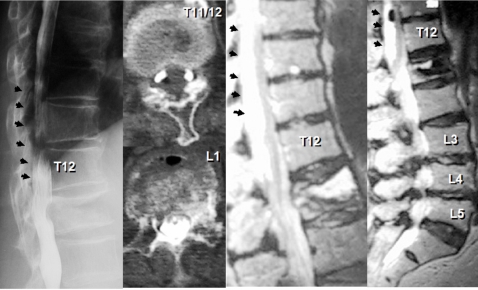
A case of a 78-year-old female. Preoperative radiographic examinations revealed L1 osteoporotic vertebral collapse with an intravertebral cleft, dorsal epidural soft tissue mass causing posterior spinal cord compression at T9-T12 levels (arrow), and severe degenerative canal stenosis at L3-L4 and L4-L5. Both intravertebral cleft and epidural soft tissue mass contained some amount of gas inside.

**Fig. (2):**
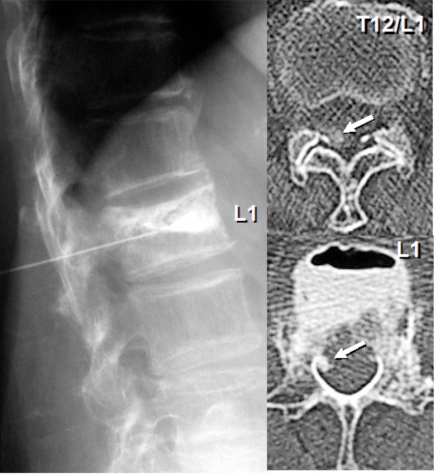
Percutaneous injection of the contrast medium into the intra-vertebral cleft showed localized enhancement of the epidural mass, indicating continuity between the intra-vertebral cleft and epidural mass.

The plain X-rays demonstrated L1 vertebral collapse with an intra-vertebral cleft. The MRIs showed dorsal epidural soft tissue mass that caused posterior compression of the spinal cord at T9-T12 levels. Continuity was observed between the intra-vertebral cleft and epidural soft tissue mass on the MRIs, and both contained some amount of gas inside. The MRIs also showed severe degenerative canal stenosis at L3-L4 and L4-L5 (Fig. **1**). Percutaneous injection of the contrast medium into the L1 intra-vertebral cleft showed localized enhancement of the epidural mass, indicating continuity between the intra-vertebral cleft and epidural mass (Fig. **2**).

**Fig. (3):**
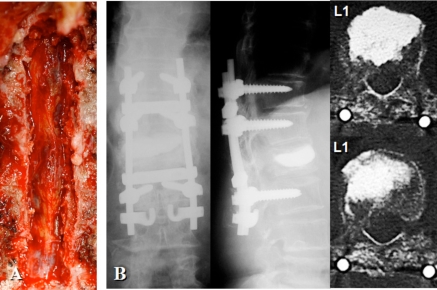
Epidural soft tissue mass was removed following laminectomy at T10-T11 and partial laminectomy at T9 and T12. The epidural mass was surrounded by a capsule and fibrous tissue, and contained coagulated blood and reddish brown fluid (**A**). Posterolateral fusion using segmental instrumentation at T11-L2 and L1 vertebroplasty was carried out (**B**).

**Fig. (4):**
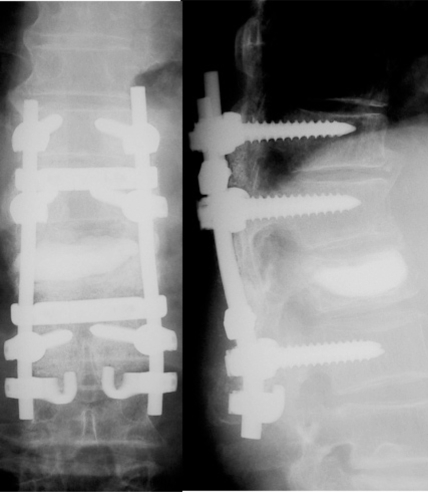
At postoperative 12 months, posterolateral bony fusion was obtained and back pain as well as neurologic symptoms has been completely solved.

One-stage posterior surgery was conducted to cure lumbar canal stenosis, thoracic epidural hematoma, and L1 vertebral collapse. First, posterior decompression at L3-4 and L4-L5 was performed. Laminectomy at T10-T11 and partial laminectomy at T9 and T12 were then carried out and followed by removal of the epidural soft tissue mass. The mass was surrounded by a capsule and fibrous tissue, and contained coagulated blood and reddish brown fluid (Fig. **3**). The soft tissue mass was macroscopically diagnosed as a chronic epidural hematoma. Posterior segmental instrumentation (M8, Medtronic Sofamor-Danek, Memphis, TN) was performed at T11-L2. Transpediclar vertebroplasty was performed using the calcium phosphate cement (Biopex R, Mitsubishi Materials Inc., Tokyo, Japan). After decortication of the posterolateral fusion bed, a mixture of local bone and bone graft substitute (Ceratite, Nippon Tokusyu Togyo, Tokyo, Japan) was grafted. The operation time was 240 minutes and intraoperative blood loss was 571g. The patient was allowed to ambulate at postoperative five days. A brace was used for two months after surgery. At postoperative 12 months, posterolateral bony fusion was obtained, back pain and neurological symptoms were completely solved, and she was able to walk without a cane (Fig. **4**).

## DISCUSSION

Spinal epidural hematoma is mostly spontaneous, post-surgical, or post-traumatic including spinal fracture [1-6]. Osteoporotic vertebral collapse is a kind of spinal fractures; however, no report to date has documented myelopathy caused by epidural hematoma associated with osteoporotic vertebral collapse. It is inferred that osteoporotic vertebral collapse rarely causes epidural hematoma because osteoporotic vertebral collapse is not an acute phase of fractures [9-11].

Kerslake RW *et al.* reported that MRIs taken within three weeks after spinal trauma demonstrated epidural hematoma in 17 of the 44 patients. However, MRIs taken more than three weeks after trauma did not indicate epidural hematoma in any patients [12]. Therefore, it is inferred that epidural hematomas caused by spinal trauma are resolved within three weeks after trauma in most cases. The current case presented epidural hematoma two months after the onset of fracture. Therefore, the hematoma could not have arisen at the time of injury. Why did the present case demonstrate epidural hematoma more than two months after the onset of vertebral fracture? One of the reasons may be that there was a connection between intra-vertebral cleft and epidural space. In other words, the fluid including hemorrhage inside the intra-vertebral cleft may have been squeezed out to the epidural space during daily motion and resulted in chronic epidural hematoma.

It is widely recognized that osteoporotic vertebral collapse sometimes causes neurological impairment [9-11]. The common causes of neurological impairment are segmental instability, kyphotic deformity, and neural compression caused by bony fragments. These can be diagnosed using plain X-rays and CTs. However, MRIs or myelograms are required if the neural compression is caused by epidural hematoma. Importantly, epidural hematoma can be a cause of neural compression if the patient develops neurological impairment during the follow-up without any radiographic changes on X-rays or CTs.

Anterior column reconstruction is necessary for osteoporotic vertebral collapse with damaged anterior column [13] and anterior decompression and reconstruction is reasonable. However, vertebral collapse combined with posterior neural compression including epidural hematoma and degenerative spinal canal stenosis, as in the current case, cannot be treated by anterior surgery alone. However, a combination of vertebroplasty and posterior decompression provides vertebral body reconstruction and posterior decompression *via *a single posterior approach. In cases of vertebral posterior wall disruption, the authors would like to recommend posterior instrumentation in conjunction with vertebroplasty to avoid migration of the CPC into the canal. Since vertebroplasty is a kind of minimally invasive procedure [14], a combination of vertebroplasty and posterolateral fusion is as safe as posterolateral fusion alone. Therefore, it can be recommended for elderly patients with osteoporotic vertebral collapse causing neural compression.

## CONCLUSIONS

A very rare case of spinal chronic epidural hematoma associated with osteoporotic vertebral collapse is reported. Although osteoporotic vertebral collapse is a kind of pseudarthorosis, it can cause chronic epidural hematoma. It was successfully treated by one-stage posterior surgery including removal of hematoma following laminectomy, vertebroplasty combined with posterior spinal reconstruction, and posterior decompression for accompanying degenerative lumbar canal stenosis.
